# Advances in the Signaling Pathways Downstream of Glial-Scar Axon Growth Inhibitors

**DOI:** 10.3389/fncel.2020.00174

**Published:** 2020-07-02

**Authors:** Armin Sami, Michael E. Selzer, Shuxin Li

**Affiliations:** ^1^Shriners Hospitals Pediatric Research Center, Lewis Katz School of Medicine at Temple University, Philadelphia, PA, United States; ^2^Department of Anatomy and Cell Biology, Lewis Katz School of Medicine at Temple University, Philadelphia, PA, United States; ^3^Department of Neurology, Lewis Katz School of Medicine at Temple University, Philadelphia, PA, United States

**Keywords:** scar inhibition, CSPG receptor, LAR, PTPσ, axon regeneration, intracellular signaling, RhoA, therapeutic target

## Abstract

Axon growth inhibitors generated by reactive glial scars play an important role in failure of axon regeneration after CNS injury in mature mammals. Among the inhibitory factors, chondroitin sulfate proteoglycans (CSPGs) are potent suppressors of axon regeneration and are important molecular targets for designing effective therapies for traumatic brain injury or spinal cord injury (SCI). CSPGs bind with high affinity to several transmembrane receptors, including two members of the leukocyte common antigen related (LAR) subfamily of receptor protein tyrosine phosphatases (RPTPs). Recent studies demonstrate that multiple intracellular signaling pathways downstream of these two RPTPs mediate the growth-inhibitory actions of CSPGs. A better understanding of these signaling pathways may facilitate development of new and effective therapies for CNS disorders characterized by axonal disconnections. This review will focus on recent advances in the downstream signaling pathways of scar-mediated inhibition and their potential as the molecular targets for CNS repair.

## Introduction

**Figure 1:**
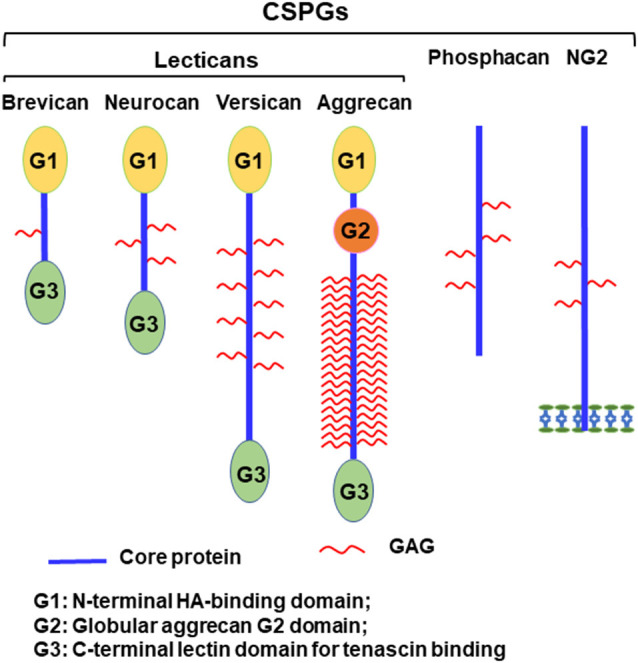
Schematic of chondroitin sulfate proteoglycans (CSPG) molecules expressed in the CNS. Lecticans include a core protein with globular G1 (N-terminal) and G3 (C-terminal) domains linked by a central domain to the chondroitin sulfate glycosaminoglycan (CS-GAG) chains. Unlike other lecticans, aggrecan also has a G2 domain linked to G1 by an interglobular domain. The G1 domain binds to hyaluronan and to link proteins, while the G3 domain interacts with tenascins and glycolipids through a lectin-like region. Phosphacan is a secreted splice variant of transmembrane receptor-type protein tyrosine phosphatase β (PTPβ). NG2 is a transmembrane proteoglycan and exists in a soluble form after its proteolytic cleavage.

Axonal disconnections in the CNS usually result in permeant dysfunction. Although glial scars play supportive role in tissue repair (Anderson et al., 2016), eventually they create an inhibitory environment for axon regeneration and contribute to functional loss after CNS injuries (Bradbury et al., 2002; Jones et al., 2003; Busch and Silver, 2007). Glial scars not only form a physical barrier but also upregulate chondroitin sulfate proteoglycans (CSPGs) and other extracellular matrix (ECM) molecules, which potently inhibit regrowth of injured axons into and beyond the lesion (Bradbury et al., 2002; Jones et al., 2003; Busch and Silver, 2007). CSPGs are strongly expressed in the lesion penumbra, with even higher levels in the epicenter of the scar (Davies et al., 1997). CSPGs are mostly produced by reactive astrocytes and to a lesser extent by other cells, including oligodendrocytes and monocytes. CSPGs are also produced and secreted by neurons in the developmental and adult CNS (Galtrey and Fawcett, 2007; Dyck and Karimi-Abdolrezaee, 2015). The major CSPGs expressed in the CNS include lecticans (aggrecan, brevican, neurocan, and versican), phosphacan, and NG2 (Figure 1; Bandtlow and Zimmermann, 2000). Lecticans have similar N-terminal hyaluronan-binding domains and C-terminal globular domains with a unique lectin domain. Lectican core proteins (size: 97–262 kD) are connected by a dominant chondroitin sulfate glycosaminoglycan (CS-GAG) anchoring backbone, bound to one or more long-chain CS-GAG polysaccharides (Yu et al., 2017). Lecticans interact with carbohydrate and protein ligands in the ECM to form the perineuronal nets (PNNs; Yamaguchi, 2000), which are primarily composed of hyaluronan, CSPGs, tenascin R, and link proteins. Interactions among these molecules form a stable pericellular complex around synapses. PNNs are formed during late stages of CNS development and probably are essential for the reduction of plasticity seen in mature neurons (Kwok et al., 2011). Phosphacan is the extracellular domain of the transmembrane receptor-type protein tyrosine phosphatase β (PTPβ). NG2 is a transmembrane CSPG that has no strong homologies to other proteins. CSPGs also are expressed in other systems and mediate proliferation, adhesion, migration, and growth of various cell types, such as neoplastic, immune ad cartilage cells.

**Figure 2:**
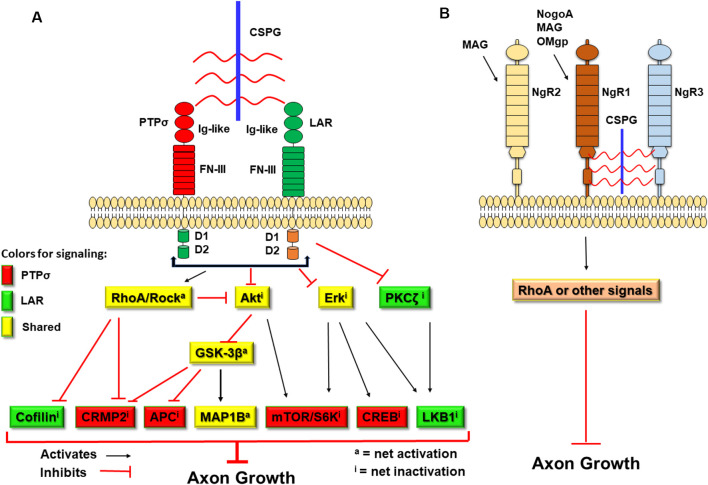
Schematic of the transmembrane receptors for CSPGs and myelin-associated inhibitors and the signaling pathways downstream of two receptor protein tyrosine phosphatases (RPTPs). CSPGs induce growth inhibition by binding and activating several transmembrane receptors, including PTPσ, leukocyte common antigen related (LAR), NgR1, and NgR3. **(A)** Intracellularly, activation of PTPσ and LAR by CSPGs activate RhoA/ROCK signaling and inactivate Akt and Erk pathways. However, the two receptors use distinct pathways downstream of RhoA/ROCK, Akt/GSK-3β, and Erk signals to mediate inhibition of axon growth by CSPGs. CSPGs also can bind NgR1 and NgR3 to inhibit axon growth. Intracellularly, NgR functions are mediated mainly by the Rho pathway. Red-filled: signals conveyed by PTPσ. Green-filled: signals conveyed by LAR. Yellow-filled: signals shared by both PTPσ and LAR. Ig-like, immunoglobulin-like domains; FN-III, fibronectin Type III domains; D1, D1 domain; D2, D2 domain; S6K, S6 kinase. **(B)** Myelin-associated inhibitors (NogoA, MAG, and OMgp) interact with NgR1 (for all three) and NgR2 (for MAG) to suppress axonal growth through RhoA activation or other pathways.

CSPGs have been known to inhibit axonal growth for 30 years (Snow et al., 1990; McKeon et al., 1991), but the molecular mechanisms underlying their actions are not fully understood (Sharma et al., 2012; Ohtake and Li, 2015). All CSPGs have similar molecular structures, including GAG chains attached to the core proteins (Figure 1; Bandtlow and Zimmermann, 2000). Because digestion of GAGs by bacterial chondroitinase ABC (ChABC), or prevention of GAG sulfation, largely reduces suppression of axon growth by CSPGs (Gilbert et al., 2005; Sherman and Back, 2008; Wang et al., 2008), sulfated GAG chains are particularly important for the inhibitory properties of CSPGs. Previously, CSPGs were shown to block growth by sterically hindering growth-promoting adhesion molecules (such as laminins/integrins) and/or facilitating suppression by repulsive molecules, such as Sema 3a and 5a (Condic et al., 1999; Kantor et al., 2004; Tan et al., 2011). During the past decade, several groups demonstrated the crucial roles of some transmembrane receptors in mediating CSPG inhibition. Two members of the LAR (leukocyte common antigen related) subfamily of receptor protein tyrosine phosphatases (RPTPs), PTPσ and LAR, bind CSPGs with high affinity and mediate suppression of axon elongation by CSPGs (Figure 2; Shen et al., 2009; Fisher et al., 2011). Nogo receptor 1 (NgR1) and NgR3, two receptors for myelin associated inhibitors, also interact with CSPGs and may mediate some of their actions (Dickendesher et al., 2012).

The intracellular signals downstream of CSPGs are not completely known, but a number of signaling proteins at least in part mediate CSPG inhibition on neuronal growth, including Akt, glycogen synthase kinase 3β (GSK-3β), RhoA, and Erk (Powell et al., 2001; Monnier et al., 2003; Sivasankaran et al., 2004; Fu et al., 2007; Dill et al., 2008). Importantly, as the crucial functional receptors of CSPGs from the same LAR subfamily (Shen et al., 2009; Fisher et al., 2011), PTPσ and LAR regulate neuronal functions by both convergent and divergent pathways (Ohtake et al., 2016). Several drugs that target the intracellular signals of CSPGs and other inhibitors have been moved to clinical trials for treating CNS axonal injuries (Kim et al., 2017; Wang et al., 2019). In this review, we focus on the progress that has been made recently in identifying CSPG signaling pathways and on their potential as the molecular targets for treating CNS injuries (Table 1), primarily in mammalian models. However, in mammals, it is difficult to do experiments with complete spinal cord transections and to distinguish regeneration of severed axons from collateral sprouting by spared fibers. Because these two processes may have different mechanisms (Jin et al., 2009), cautious investigators often refer to “axon growth” or “plasticity” rather than “regeneration” (Blesch and Tuszynski, 2009). Therefore, in some cases, we cite relevant work in non-mammalian species, particularly lampreys, which show robust but only partial axon regeneration after complete spinal cord transection, upregulate CSPGs at the injury site, and express homologs of the most molecules referred to in this review.

**Table 1:** Summary of the major topics reviewed in this article.

Major topics reviewed	Critical information on the topic
Neuronal receptors for CSPGs	• PTPσ, LAR, NgR1, and NgR3
Potential intracellular signals to convey CSPG inhibition	• RhoA/ROCK, Akt/ GSK-3β, PKC, and MARK
Comparisons of the signals downstream of PTPσ and LAR	Shared signals by both receptors: RhoA, Akt/GSK-3β, Erk, and MAP1B Major signals downstream of PTPσ: CRMP2, APC, mTOR/S6 kinase, and CREB Major signals downstream of LAR: cofilin, PKCζ and LKB1
Clinical trial drugs to target CSPG downstream signals	RhoA inhibitors: cell-permeable C3 (also called Cethrin, BA-210 and VX-210) and high dose of ibuprofen GSK-3 inhibitor: lithium

## Several Transmembrane Receptors Mediate Growth Suppression of CSPG Inhibitors

The unique structures of CSPGs are essential for their function because digesting their GAG chains with enzymes, or preventing sulfation of GAG chains, greatly reduces their inhibition of axon growth (Gilbert et al., 2005; Sherman and Back, 2008; Wang et al., 2008; Brown et al., 2012). CSPGs are characterized by a core protein to which the highly sulfated GAG chains are attached (Figure 1). Previous studies suggested that CSPGs acted through non-specific steric interactions, hindering the binding of ECM molecules to their cell surface receptors. Interactions between laminins and their receptor integrins, the growth-promoting adhesion ECM molecules, are crucial for regulating axon growth. CSPGs interrupted the laminin-integrin interactions and attenuated integrin activation (Condic et al., 1999; Afshari et al., 2010). Accordingly, high levels of integrins overcame growth inhibition by CSPGs (Tan et al., 2011).

CSPGs potentiate the functions of some repulsive proteins, such as Sema5A and Sema3A. The GAGs of both CSPGs and heparan sulfate proteoglycans (HSPGs) interacted with the thrombospondin repeats of Sema5A, and CSPG binding could convert Sema5A from an attractive to an inhibitory guidance cue (Kantor et al., 2004). The CS-E motifs of CSPGs, which are enriched in the perineuronal nets, could also interact with Sema3A and contribute to the chemo-repulsive action of this guidance cue (De Wit et al., 2005; Deepa et al., 2006; Kwok et al., 2011). Moreover, the CSPG GAGs could block neuronal growth by binding extracellular calcium or its channels, limiting calcium entry into neurons (Hrabetova et al., 2009).

Despite the molecular mechanisms described above, several transmembrane receptors are important in conveying CSPG inhibition of axon growth. Out of three vertebrate homologs (LAR, PTPσ, and PTPδ) in the LAR subfamily, LAR and PTPσ have been shown to be neuronal transmembrane receptors essential for mediating growth suppression by CSPGs. CSPG GAG chains bind several positively charged amino acids in the first Ig-like domain of PTPσ and LAR and activate these phosphatases (Aricescu et al., 2002; Shen et al., 2009; Fisher et al., 2011). Consistently, a PTPσ homolog binds the GAG chains of the HSPG agrin and of collagen XVIII also by its first Ig-like domain, but promotes retinal axon growth (Ledig et al., 1999; Aricescu et al., 2002). *Drosophila* LAR binds to the HSPGs syndecan and dallylike with high affinity, and thereby regulates synaptic function (Fox and Zinn, 2005; Johnson et al., 2006). A further study demonstrates that HSPGs and CSPGs compete for the same binding site on the first Ig domain of PTPσ (Coles et al., 2011). Because HSPG binding triggers PTPσ oligomerization and CSPG binding has the opposite effect, the ratio of CSPG:HSPG determines the overall activation status of this receptor. Upregulation of CSPGs blocks PTPσ oligomerization, activates this receptor, and thus suppresses neuronal outgrowth. Therefore, PTPσ is a bifunctional receptor and its activity depends on the types of ligands bound to it.

PTPσ and LAR are important functional receptors for CSPGs in adult mammals. In neuronal cultures, deletion of either PTPσ or LAR overcomes growth inhibition by CSPGs, but not by myelin associated inhibitors (Shen et al., 2009; Fisher et al., 2011). Deficiency of either PTPσ or LAR significantly increased regrowth of corticospinal tract neurons into the spinal cord several millimeters caudal to the lesion in adult mice with mid-thoracic hemisection injury (Fry et al., 2010; Fisher et al., 2011). Suppressing PTPσ or LAR also stimulated regrowth of other spinal cord tracts after spinal cord injury (SCI), including sensory (Shen et al., 2009) and serotonergic axons (Fisher et al., 2011; Lang et al., 2015). Previous studies had reported that regeneration of injured optic nerve and peripheral nerves was enhanced in PTPσ knockout mice (McLean et al., 2002; Thompson et al., 2003; Sapieha et al., 2005; Fry et al., 2010). It is not yet known whether PTPδ, the third member in LAR subfamily, also acts as a CSPG receptor to mediate inhibition of axon regeneration. PTPδ mediated Sema3A-regulated neuronal growth by activating Fyn and Src kinases (Nakamura et al., 2017). Similar to PTPσ and LAR, PTPδ regulates synaptogenesis during development and PTPδ variants bind with nanomolar affinities to recombinant versions of the HSPG glypican-4 (Ko et al., 2015).

Both LAR and PTPσ are important therapeutic targets to promote CNS axon regeneration in adult mammals. Pharmacological blockade of either LAR or PTPσ after SCI significantly promotes motor axon regrowth and functional recovery in adult rodents. Systemic treatments with small peptides representing extracellular or intracellular LAR sequences increased the density of serotonergic fibers in spinal cord 5–7 mm caudal to the lesion in adult mice with T7 dorsal over-hemisection, and also promoted recovery of locomotor function, as determined by multiple behavioral tests (Fisher et al., 2011). Similarly, systemic delivery of a peptide representing the intracellular PTPσ sequence dramatically enhanced regrowth of serotonergic axons into the caudal spinal cord, and promoted functional recovery in both locomotor and urinary systems of adult rats with thoracic contusion SCI (Lang et al., 2015).

In lampreys, both LAR and PTPσ are expressed selectively in neurons that regenerate poorly post-axotomy (Zhang et al., 2014). Paradoxically, knockdown of PTPσ by retrograde delivery of morpholinos from the transection site was followed by inhibition of regeneration and reduction in some measures of locomotor recovery (Rodemer et al., 2020). Presumably, PTPσ plays more than one role in the nervous system and the net effect of its knockdown may depend on the balance among its several roles in a given species and environment. In these lamprey experiments, the morpholino also enterred local cells at the lesion site, so the effect of PTPσ knockdown might be indirect through actions extrinsic to the reticulospinal neurons. This may highlight the difficulties in translating *in vitro* studies to partial SCI models and the effects of genetic manipulations to therapies for human SCI.

NgR1 and NgR3 also act as receptors to mediate growth suppression by CSPGs (Figure 2). The NgR family includes three GPI-anchored receptors (NgR1, NgR2, and NgR3), which have similar structures, including the eight leucine-rich repeats (LRR) flanked by N-terminal and C-terminal LRR-capping domains. NgR1 is the receptor for three myelin associated inhibitors: NogoA, myelin associated glycoprotein (MAG), and oligodendrocyte myelin glycoprotein (OMgp; Fournier et al., 2001, 2002; McGee and Strittmatter, 2003; Liu et al., 2006), while NgR2 binds MAG but not MAP and OMgp (Venkatesh et al., 2005). The ligands that bind NgR3 have not been fully identified. NgR1 and NgR3 interacted with CS and dermatan sulfate chains and functioned as receptors for these proteoglycans (Dickendesher et al., 2012). In this study, deletion of both NgR1 and NgR3, but not NgR1 and NgR2, overcame CSPG inhibition and promoted regeneration of injured optic nerves in double knockout mice, suggesting that NgR1 and NgR3 mediate some suppression by two completely different groups of inhibitors generated by oligodendrocytes and reactive astrocytes. Furthermore, NgR2 bound versican at the dermo-epidermal junction and this interaction regulated plasticity of peripheral sensory fibers (Bäumer et al., 2014).

## Numerous Intracellular Signals Convey CSPG Inhibition on Neuronal Growth

Several intracellular signals have been shown to mediate CSPG inhibition on neuronal growth, including RhoA, Akt, GSK-3β, protein kinase C (PKC), and others (Ohtake and Li, 2015). CSPGs, myelin-derived growth inhibitors, and repulsive guidance molecules converge on certain downstream signals, such as activation of RhoA and inactivation of Akt, to regulate neuronal growth (Figure 2; Luo, 2000; Etienne-Manneville and Hall, 2002; McGee and Strittmatter, 2003; Mueller et al., 2005; Fu et al., 2007; Dill et al., 2008; Hata et al., 2009). Targeting the converged downstream signals, e.g., inhibiting the Rho signaling pathway, can overcome growth suppression by CSPGs and other inhibitors, thereby promoting axon elongation.

RhoA is important in mediating inhibition of axon growth by CSPGs, myelin-associated inhibitors, and other repulsive guidance cues. In addition to myelin inhibitors (NogoA, MAG, and OMgp; Niederöst et al., 2002) and guidance cues (Sema 3a and Ephrin; Klein, 2004; Gallo, 2006), CSPGs increase activities of RhoA and its effector Rho-associated kinase (ROCK) in neurons (Fournier et al., 2003; Monnier et al., 2003; Jain et al., 2004; Fu et al., 2007; Dill et al., 2008). CSPGs also control growth by targeting local RhoA in the axons because applying CSPGs to the axonal compartment of microfluidic chambers increased intra-axonal RhoA synthesis, while depleting RhoA transcripts in axons promoted their growth in the presence of CSPGs (Walker et al., 2012).

Rho and ROCK act as central regulators of axonal growth by targeting numerous growth-related proteins. Activating the Rho/ROCK pathway phosphorylates several downstream proteins (such as LIM domain kinase 2 and myosin light chain) promotes F-actin disassembly in axonal growth cones (Maekawa et al., 1999), prevents recruitment of microtubules required for axon elongation (Rodriguez et al., 2003), and subsequently induces growth cone collapse by rearranging cytoskeletal proteins (Maekawa et al., 1999; Wu et al., 2005). Collapsin response mediator protein 4 (CRMP4), a microtubule interacting protein, involves CSPG inhibition because CRMP4 deletion promotes growth of neurites on CSPGs *in vitro* and elongation of sensory axons after spinal cord transection (Nagai et al., 2016). Activation of ROCK by CSPGs and MAG increases the turnover of α-tubulin acetyltransferase-1α, which acetylates α-tubulin and thus reduces the levels of this acetyltransferase post-transcriptionally (Wong et al., 2018). Therefore, blocking either RhoA or ROCK with inhibitors overcomes growth inhibition by CSPGs and other inhibitors (Borisoff et al., 2003; Mueller et al., 2005) and enhances axon regeneration and functional recovery after CNS axon injury in rodents (Fournier et al., 2003). Moreover, knockdown of RhoA in lamprey reticulospinal neurons by retrogradely-delivered morpholinos inhibited retrograde apoptotic signaling and axon retraction and promoted axon regeneration after SCI (Hu et al., 2017). There also was evidence for transient upregulation of RhoA mRNA in these neurons and for its rapid translocation to the axon tip. After 2 weeks, RhoA expression was reduced selectively in those reticulospinal neurons that did not show caspase activity (Zhang et al., 2018).

CSPGs inactivate PI3K/Akt signals, another intracellular pathway essential for controlling neuronal growth (Ohtake and Li, 2015). In neuronal cultures, CSPG stimulation reduces the levels of phosphorylated Akt at Ser473 and S6 kinase (Dill et al., 2008; Fisher et al., 2011; Silver et al., 2014; Ohtake et al., 2016). Inactivation of Akt is likely to suppress signaling by mTOR, an upstream activator of protein synthesis involved in axonal regeneration (Park et al., 2008, 2010; Liu et al., 2010). Accordingly, deleting PTEN and degrading CSPGs prevent mTOR inhibition and enhance axon regrowth after CNS injury (Lee et al., 2014). Application of CSPGs also enhances the levels of 4E-BP1 (Walker et al., 2012), a negative signal along the PI3K/Akt pathway (Yang et al., 2014).

PKC also appears to be a downstream signal to mediate CSPG action. CSPGs and myelin-associated inhibitors activated PKC, while PKC blockade overcame the growth suppression and promoted regeneration of dorsal column sensory axons in adult rats with SCI (Sivasankaran et al., 2004). However, application of CS-E tetrasaccharide, one of the CSPG GAG chains, enhanced neurite outgrowth, and inhibition of PKC with bisindolylmaleimide II or digestion of GAGs with ChABC abolished the increased axon growth (Sotogaku et al., 2007). Thus, further studies are required to dissect the role of PKC in mediating CSPG action.

CSPGs also activate epidermal growth factor receptor (EGFR) pathways, while suppressing the kinase activity of EGFR, or its downstream mitogen-activated protein kinase (MAPK), reverses inhibition of axon growth by CSPGs (Koprivica et al., 2005; Kaneko et al., 2007). Inhibition of ErbB1, an EGFR, by either pharmacological or genetic approach, promotes neuronal growth in the presence of CSPG, CNS myelin, or fibrinogen (Leinster et al., 2013). Moreover, myosin II, an ATP-dependent motor protein, probably mediates CSPG inhibition on axonal growth (Hur et al., 2011; Yu et al., 2012). CSPGs increase phosphorylation of nonmuscle myosin II regulatory light chains, while pharmacological or genetic inhibition of myosin II promotes axon growth *in vitro* on inhibitory substrates including CSPGs and axon regeneration after optic nerve injury (Wang et al., 2020).

Repulsive guidance molecule A (RGMa), a GPI-linked glycoprotein, inhibits axon growth by interacting with its neuronal receptor neogenin, but it also promotes reactive astrogliosis and glial scar formation by activating the TGFβ1-Smad2/3 signaling pathway and preventing neurological functional recovery after stroke (Zhang et al., 2018). Consistently, treatments with RGMa antibodies promoted axon regeneration and functional recovery in adult rodents with SCI and also showed a trend toward reducing CSPG expression around the lesion (Hata et al., 2006; Mothe et al., 2017). In lampreys, neogenin mRNA is expressed selectively in poorly regenerating reticulospinal neurons, while RGM is downregulated in cells near a spinal cord transection, possibly increasing the ability of axons to grow through the lesion (Shifman et al., 2009).

## Two RPTPs Use Both Convergent and Divergent Signaling Pathways to Convey CSPG Inhibition in Neurons

CSPGs may suppress neuronal growth by sterically hindering growth-promoting adhesion molecules and facilitating inhibition by chemo-repulsive molecules (Condic et al., 1999; Kantor et al., 2004; Tan et al., 2011), but some receptors are important in transmitting CSPG inhibition, including PTPσ, LAR, NgR 1, and NgR3 (Shen et al., 2009; Fisher et al., 2011; Dickendesher et al., 2012; Lang et al., 2015; Xu et al., 2015). Among them, PTPσ and LAR, two members in the LAR subfamily, bind CSPGs with high affinity and mediate their suppression of neuronal growth. Using combined gain-of-function and loss-of function approaches (Ohtake et al., 2016), a group recently demonstrates that LAR and PTPσ use several common signaling pathways, including RhoA, Akt, Erk, and MAP1B (Ohtake et al., 2016). But they also transmit CSPG effects with distinct signals, including the use of CRMP2, adenomatous polyposis coli (APC), S6 kinase and CREB by PTPσ, and the use of cofilin, PKCζ, and liver kinase B1 (LKB1) by LAR (Figure 2). Combined deficiencies of both receptors promoted additional neurite outgrowth by adult neurons in the presence of CSPGs *in vitro*.

RhoA signaling mediates functions of both LAR and PTPσ in neurons. Application of CSPGs significantly increases the levels of active RhoA in N2A cells (a neuronal cell line) transfected with either PTPσ or LAR, indicating that interactions between CSPGs and each of the PTPs separately activate RhoA (Ohtake et al., 2016). Consistently, administration of CSPGs increased the levels of active RhoA in cultured cerebellar granule neurons (CGNs) derived from wildtype (WT) mice, but not from either PTPσ−/− or LAR−/− mice. As a downstream mediator of RhoA, ROCK can phosphorylate and activate LIM kinase, which in turn phosphorylates and inactivates cofilin (Maekawa et al., 1999; Zhang et al., 2006). Actin depolymerizing factor (ADF) and cofilin are highly expressed in the actin disassembly region of neuronal growth cones and play a critical role in regulating actin dynamics and axon growth. Upregulating ADF/cofilin increased neurite length by disassembling actin filaments and promoting microtubule-based extension. ADF/cofilin also controlled actin turnover to sustain regeneration of adult rodent axons after SCI by disarranging actin (Tedeschi et al., 2019). Levels of p-cofilin were dramatically elevated in LAR-transfected but not PTPσ-transfected N2A cells after incubation with CSPGs, suggesting that CSPG-LAR interactions increase cofilin phosphorylation and inhibit axon elongation by reducing actin disassembly. Accordingly, ROCK inhibitors diminished p-cofilin levels in CSPG-stimulated PC12 cells (Gopalakrishnan et al., 2008). CSPG stimulation also boosts the levels of p-cofilin in WT, but not LAR−/− CGNs. Therefore, inactivating cofilin by phosphorylation probably mediates the inhibition by CSPG-LAR, but not CSPG-PTPσ.

Several signals in the mTOR pathway convey the functions of both LAR and PTPσ although they mostly mediate the actions of PTPσ (Ohtake et al., 2016). CSPG stimulation reduced the levels of p-Akt in N2A cells transfected with either PTPσ or LAR, and in WT CGNs, but not in PTPσ−/− or LAR−/− CGNs, indicating that Akt mediates functions of both receptors upon CSPG application. Increased Akt activity has also been reported in PTPσ−/− neurons (Sapieha et al., 2005). Activation (phosphorylation) of S6 kinase, a downstream effector of mTOR, was reduced by CSPGs in a neuronal cell line overexpressing PTPσ, but not LAR, and in WT, but not PTPσ-deleted CGNs. Thus, S6 kinase mediates the interactions between CSPGs and PTPσ, but not LAR. The levels of pS6 have been correlated with increased translation of many mRNA transcripts and cell growth (Park et al., 2008).

Another mTOR downstream signal, p-4E-BP1(Thr37/46, inactive), plays a modest role in regulating CSPG-receptor interactions (Ohtake et al., 2016). Phosphorylation of CRMP2, a signal downstream of both the Akt and Rho pathways, contributes to axon growth inhibition produced by CSPG-PTPσ interactions. Inactivation of CRMP2 by phosphorylation induces axon growth cone collapse (Arimura et al., 2005; Uchida et al., 2005; Petratos et al., 2008; Wakatsuki et al., 2011). Furthermore, APC and MAP1B, two substrates of GSK-3β in the mTOR pathway, mediate functions of PTPσ (Figure 2), but APC is not involved in LAR action, and MAP1B appears to mediate the action of LAR to a greater degree than that of PTPσ (Ohtake et al., 2016).

Erk mediates actions of PTPσ and LAR by distinct downstream signaling pathways (Ohtake et al., 2016). Evaluation of Erk activity by measuring p-p44/42 MAPK (Erk1/2) at Thr202/Tyr204 demonstrates that CSPGs greatly reduce its levels in neurons derived from WT mice, but not PTPσ- or LAR-deleted mice. CSPGs and myelin-associated inhibitors were previously linked to Erk signaling (Stern and Knöll, 2014), and treatment with nerve growth factor, a signal upstream of Erk, promoted neuronal growth on CSPGs (Zhou et al., 2006). Downstream of Erk, CREB regulates the interactions between CSPGs and PTPσ. In contrast, the 90 kDa ribosomal S6 kinases (p90RSKs), which are characterized by two non-identical functional kinase domains and a C-terminal docking site for Erks (Fisher and Blenis, 1996; Smith et al., 1999), play only a minor role in mediating functions of PTPσ and LAR.

LKB1 signaling mediates CSPG-LAR, but not CSPG-PTPσ, interactions (Ohtake et al., 2016). Erk and S6 kinase phosphorylate multiple substrates, including LKB1 at Ser325 and Ser428 (Ser431 in the mouse), which is critical for subsequent activation of AMP-activated protein kinase (AMPK; Zheng et al., 2009) and for control of axon development (Barnes et al., 2007; Huang et al., 2014). Measurement of p-LKB1 indicated the significant role for LKB1 in mediating LAR, but not PTPσ action. PKCζ, a member of the atypical PKC subfamily, phosphorylates LKB1 at Ser-428/431 and mediates AMPK activation in endothelial cells (Shifman et al., 2009; Xu et al., 2015). In neurons, the PKCζ isoform contributes to the action of LAR in response to CSPG application, probably by regulating LKB1 activity (Ohtake et al., 2016). Overexpression of LKB1 in mature neurons promoted dramatic axon regeneration in adult mice with SCI (Ohtake et al., 2019). NG2, a non-lectican CSPG, also activates PKCζ and Cdc42 and increases association of PKCζ with Par6 (Lee et al., 2013). Although the cAMP-dependent PKA may activate LKB1 by phosphorylating it at S431, and thereby promote axon differentiation during development (Collins et al., 2000; Barnes et al., 2007; Shelly et al., 2007), PKA plays only a minimal role in mediating actions of LAR or PTPσ (Ohtake et al., 2016). Neither does the conventional PKC mediate the functions of either LAR or PTPσ in neurons.

Several other proteins have been reported to function as signals downstream of PTPσ. NDPK2 (nucleoside diphosphate kinase 2) interacts with PTPσ and NDPK2 knockdown in cortical neurons overcomes inhibition by CSPGs (Hamasaki et al., 2016). Ezrin, a linker protein between the plasma membrane and actin cytoskeleton, is a substrate of PTPσ and regulates migration and organization of cells by altering adhesion of their surface structures (Doody et al., 2015). N-cadherin and β-catenin are also PTPσ substrates and may regulate axon growth downstream of PTPσ (Siu et al., 2007). Moreover, CSPG-PTPσ interactions may regulate autophagic flux at the axon growth cone by inhibiting autophagosome-lysosomal fusion step (Tran et al., 2020).

Together, LAR and PTPσ share certain signaling pathways but also employ distinct signals to covey CSPG effects on neurons, suggesting that targeting both PTP receptors have additive effects in overcoming CSPG inhibition of axon growth. Indeed, deleting either of these receptors enhanced neurite growth in adult neurons cultured on CSPGs, but deleting both displayed greater axon growth *in vitro*, indicating synergy between two RPTPs in mediating CSPG function (Ohtake et al., 2016).

## CSPG Downstream Signals Are Therapeutic Targets for CNS Injuries

Currently, there are no FDA approved pharmacological therapies to recover motor and sensory functions in patients with CNS axon injuries. Overcoming strong suppression by glial scars and CSPG-mediated growth inhibition is a major target for therapeutic intervention after CNS injuries. Previously, the main *in vivo* method to overcome inhibition by CSPGs was enzymatic digestion with locally applied ChABC, but some disadvantages (such as the short duration of enzymatic activity at 37°C and potential immune responses after repeated applications) may prevent using this bacterial enzyme as a therapeutic option for patients (Sharma et al., 2012; Ohtake and Li, 2015). Better understanding of signaling pathways, including identification of CSPG receptors, has led to new approaches for overcoming CSPG-mediated inhibition. Systemic applications of selective antagonists for CSPG receptors have promoted remarkable axon regrowth and functional recovery of motor and autonomic systems after SCI (Fisher et al., 2011; Lang et al., 2015; Urban et al., 2019a,b). Diverse axon growth inhibitors from scar tissues and myelin debris suppress neuronal growth through converging downstream pathways, suggesting the alternative approaches to overcome inhibition of axon growth in the CNS. Some compounds that regulate the activities of intracellular signals, including inhibitors of signaling by Rho and GSK-3β, are promising for promoting CNS axon regeneration and recovery in adult mammals (Meijer et al., 2004; Mueller et al., 2005; McKerracher and Higuchi, 2006; Fehlings et al., 2011).

Inhibitors of RhoA and ROCK have been reported to promote cell survival, axon regrowth, and functional recovery after SCI, stroke, and other CNS injuries (Kubo and Yamashita, 2007; Chong et al., 2017; Sladojevic et al., 2017). After CNS lesions, CSPGs and other inhibitors activate Rho and its downstream ROCK, which cause apoptotic cell loss, growth cone collapse, and axon regeneration failure. The bacterial C3 transferase and its derivatives have been frequently used as selective inhibitors of Rho by ADP-ribosylating the Rho effector domain and blocking Rho function. C3 treatment prevents cell loss and promotes axon regeneration after SCI and optic nerve crush in rodents (Lehmann et al., 1999; Dergham et al., 2002; Dubreuil et al., 2003). In SCI rodents, a modified C3 that is cell-permeable (also called Cethrin, BA-210, and VX-210) and a recombinant fusion protein containing C3 inactivated RhoA in the lesioned spinal cord reduced secondary tissue damage and glial scarring, stimulated axon regeneration and functional recovery, even when applied 16 days after injury (Lord-Fontaine et al., 2008). Other Rho inhibitors that have been used to promote CNS regeneration include C3-peptides, siRNA, and ibuprofen (Fu et al., 2007; Boato et al., 2010; Dill et al., 2010; Gwak et al., 2017). Furthermore, several ROCK inhibitors have been developed, including Fasudil and its derivatives (hydroxyfasudil and dimethylfusudil), Y-27632 and Y-39983. They also enhanced axon regrowth and provided neuroprotection in different models of neurological disorders (Kubo and Yamashita, 2007; Fujita and Yamashita, 2014).

Because preclinical studies showed promising results on the Rho inhibitor C3, a phase I/IIa clinical trial of VX-210 had been completed to test its potential to enhance motor function in patients with acute SCI (NCT00500812; Fehlings et al., 2011; McKerracher and Anderson, 2013). In this trial, 48 acute traumatic cervical and thoracic SCI patients received a single dose of VX-210 (range: 0.3–9.0 mg) applied to the spinal cord dura mater during decompression surgery within the first 7 days after injury. The results suggested the tolerability of the treatment and improvement in motor strength in patients with cervical SCI compared to its natural course. The recovery trajectory of thoracic SCI cases was similar to that in natural history studies (Fehlings et al., 2011). Because of the promising results of phase I/IIa clinical trial, VX-210 was moved to a phase IIb/III SPRING (SCI Rho inhibition investigation) trial for acute traumatic cervical SCI (NCT02669849). The major goal of this randomized, double-blind, placebo-controlled trial was to evaluate its safety and efficacy in promoting functional recovery by Rho inhibition (Fehlings et al., 2018). However, this trial was terminated by Vertex probably because of lack of efficacy after an interim analysis.

A subset of non-steroidal anti-inflammatory drugs (NSAIDs), including ibuprofen, indomethacin, and sulindac sulfide, inhibit Rho activity independently of their classical function as the inhibitors of cyclooxygenases and promote axon regrowth and functional recovery after SCI in rodents (Zhou et al., 2003; Fu et al., 2007; Wang et al., 2009). PPARγ activation contributes to the ibuprofen-mediated Rho inhibition in neurons (Dill et al., 2010). Meta-analysis of multiple preclinical studies on the effects of Rho-inhibiting NSAIDs suggested a moderate effect on motor recovery after ibuprofen or indomethacin treatment (Watzlawick et al., 2014). Because NSAIDs are widely used clinically for relieving pain and treating various disorders, it would be especially attractive to test Rho-inhibiting NSAIDS, including ibuprofen, as treatments for CNS axonal injuries. A phase I clinical SCISSOR (SCI study on small molecule-derived Rho inhibition) trial has been initiated to treat acute SCI with high-dose ibuprofen (NCT02096913; Kopp et al., 2016). The key inclusion criteria include acute traumatic motor-complete SCI (classified as AIS A or AIS B) with lesions at the levels of C4-Th4. The major goal of this trial is to evaluate the safety, feasibility, and pharmacokinetics of ibuprofen when used at the high dose of 2,400 mg per day. Preclinical studies showed potent Rho inhibition by ibuprofen when applied at high doses (50–70 mg/Kg body weight; Zhou et al., 2003; Fu et al., 2007; Wang et al., 2009).

Treatments with GSK-3β inhibitors, including the clinical drug lithium, have overcome the growth suppression of CNS inhibitory substrates and promoted axonal regeneration and functional recovery in adult rodents with SCI (Dill et al., 2008). As one of the downstream signals in the mTOR pathway, GSK-3β mediates CSPG action and plays a critical role in regulating axon genesis and elongation. Thus, inhibiting GSK-3β, possibly with lithium, which is commonly used to treat bipolar illness, is an interesting potential therapeutic approach for promoting axon regrowth and functional recovery after CNS injuries (Dill et al., 2008; Ohtake et al., 2016). Lithium also enhanced proliferation and neuronal differentiation of neural progenitor cells in the spinal cord of adult rats (Su et al., 2007).

A phase I clinical trial was completed to evaluate the safety and pharmacokinetics of lithium in chronic SCI patients (NCT00431171). This trial suggests that lithium is safe for treating chronic SCI patients (Wong et al., 2011). A randomized, double-blind phase II clinical trial was performed to evaluate its efficacy for treating chronic SCI patients (NCT00750061; Yang et al., 2012). Lithium was effective for reducing neuropathic pain in chronic SCI but did not improve the neurological outcomes of patients. Further clinical trials with combined strategies, including transplanting umbilical cord blood-derived mononuclear cells plus oral lithium or methylprednisolone followed by locomotor training, improved both motor and autonomic functions in some SCI patients (NCT01046786 and NCT01354483; Zhu et al., 2016), but the exact role of lithium was less clear in these trials.

## Prospective

Achieving functional regeneration after CNS axonal injury is a challenging topic in neuroscience research. Although a developmentally determined reduction in the intrinsic capacity of mature neurons for growth contributes to CNS regeneration failure, the scar tissue and its associated inhibitors form potent physical and chemical barriers to axon elongation after CNS injury. Therefore, it is critical to design effective regenerative strategies that target the inhibitory CNS environment. Early after injury, reactive astrocytes exert positive effects in promoting tissue repair and axon regrowth by limiting tissue damage, preventing extension of the injury into adjacent areas, and generating many ECM components such as fibronectin and laminin that have growth-promoting properties (Bush et al., 1999; Faulkner et al., 2004; Anderson et al., 2016).

In the past decade, researchers have made significant progress in further understanding the functions of reactive scar tissue, including identification of several functional receptors for scar-associated inhibitors and dissection of intracellular signals downstream of these inhibitors. It would be valuable to identify additional receptors of CSPGs and to determine whether different CSPGs and HSPGs have binding preferences to the diverse receptors recognized. Although CSPGs, HSPGs, Ezrin, and nucleoside diphosphate kinase 2 have been recognized as the ligands of PTPσ and/or LAR, additional substrates might to be identified.

CSPGs and other extracellular molecules (such as myelin-associated inhibitors, guidance cues, and neurotrophins) share certain intracellular signals (e.g., RhoA, Akt, and Erk) that mediate neuronal growth. It would be valuable to determine whether specific interventions of the identified downstream signals alter the responses of individual extracellular molecules and their corresponding receptors. As outlined in Figure 2, PTPσ and LAR mediate CSPG actions by both convergent and divergent signaling pathways, but it is critical to dissect additional pathways, their molecular links to transmembrane receptors and cytoskeletal structures, and the potential interactions among these intracellular signals. A better understanding of the molecular pathways for axon growth regulators should facilitate the design of effective regenerative and reparative strategies to treat CNS injuries.

Overcoming strong suppression of axon growth by CSPGs and glial scars is a main target for therapeutic intervention after CNS injuries. Many researchers have focused on digestion of the CSPG GAG side chains with local application of ChABC. Because several disadvantages may prevent using this bacterial enzyme as a therapeutic option for patients, alternative approaches to neutralizing growth inhibition by glial scars are needed, including targeting CSPG receptors and their downstream signals. Indeed, intervening in the convergent signals is promising because they may convey actions of multiple extracellular molecules, including both repulsive and attractive factors for neuronal growth. Several drugs that block Rho or GSK-3β were moved to clinical trials for treating CNS axonal injuries but have not yet borne fruit. Because multiple factors contribute to CNS repair failure, combined strategies, such as effective neuroprotectants to prevent cell loss, regenerative approaches to target both neuron-intrinsic and extrinsic factors, cell/biomaterial transplants to bridge rostral and caudal neural tissues, and task-specific rehabilitative training to enhance plasticity of neural circuits, may be required to further promote neural regeneration and functional recovery after CNS injuries.

## Author Contributions

All authors listed have made a substantial, direct and intellectual contribution to the work, and approved it for publication.

## Conflict of Interest

The authors declare that the research was conducted in the absence of any commercial or financial relationships that could be construed as a potential conflict of interest.
